# Intrinsic aerobic capacity modulates Alzheimer’s disease pathological hallmarks, brain mitochondrial function and proteome during aging

**DOI:** 10.1007/s11357-024-01248-3

**Published:** 2024-06-13

**Authors:** Benjamin A. Kugler, Colton R. Lysaker, Edziu Franczak, Brittany M. Hauger, Vivien Csikos, Julia A. Stopperan, Julie A. Allen, John A. Stanford, Lauren G. Koch, Steven L. Britton, John P. Thyfault, Heather M. Wilkins

**Affiliations:** 1 University of Kansas Alzheimer’s Disease Center and Department of Neurology, Kansas City, KS USA; 2 University of Kansas Medical Center Department of Cell Biology and Physiology and Internal Medicine, Kansas City, KS USA; 3 Department of Physiology and Pharmacology, College of Medicine and Life Sciences, The University of Toledo, Toledo, OH USA; 4 Department of Anesthesiology, University of Michigan, Ann Arbor, MI USA; 5 Department of Molecular & Integrative Physiology, University of Michigan, Ann Arbor, MI USA; 6 Research Service, Kansas City VA Medical Center Department of Veterans Affairs, University of Kansas Diabetes Center, Kansas City, KS USA; 7 University of Kansas Medical Center Department of Molecular Biology and Biochemistry, Kansas City, KS USA; 8 Department of Neurology University of Kansas Medical Center, Kansas City, KS USA

**Keywords:** Mitochondria, Amyloid beta, Tau, Bioenergetics, Alzheimer’s disease, Aerobic capacity, Aging

## Abstract

Low aerobic capacity is strongly associated with all-cause mortality and risk for Alzheimer’s disease (AD). Individuals with early dementia and AD have lower aerobic capacity compared to age-matched controls. The mechanism by which aerobic capacity influences AD risk is unknown but is likely mediated by sexual dimorphism and tissue-level differences in mitochondrial energetics. Here, we used rats selectively bred for large differences in intrinsic aerobic exercise capacity. Brain tissue from 18-month and 24-month-old female and male low-capacity runner (LCR) and high-capacity runner (HCR) rats were analyzed for markers of mitochondrial function and AD-associated pathologies. LCR rats, irrespective of sex, exhibited a greater increase in brain amyloid beta (Aβ_42_) and tau hyperphosphorylation (pTau^thr181^/total tau) with aging. In female LCR rats, brain mitochondrial respiration at states 3, 4, and FCCP-induced uncoupling, when stimulated with pyruvate/malate, was reduced at 18 and 24 months, leading to lower ATP-linked mitochondrial respiration compared to mitochondria from HCR rats. Male LCR rats also showed reduced complex II-stimulated mitochondrial respiration (succinate + rotenone) at 24 months compared to HCR rats. Differences in mitochondrial respiration were associated with tau hyperphosphorylation and Aβ42 alterations in both HCR and LCR strains. Proteomic analysis unveiled a distinct difference in the mitochondrial proteome, wherein female LCR rats displayed diminished mitochondrial translation and oxidative phosphorylation (OXPHOS) proteins at 18 months compared to female HCR rats. Conversely, male LCR rats exhibited increased OXPHOS protein abundance but reduced tricarboxylic acid (TCA) cycle proteins compared to male HCR rats. These findings underscore a robust association between intrinsic aerobic exercise capacity, brain mitochondrial function, and AD pathologies during aging.

## Introduction

Alzheimer’s disease (AD) is the most common form of dementia diagnosed at autopsy with neuropathological examination [1, 2]. The pathological hallmarks that lead to AD diagnosis postmortem are amyloid beta (Aβ) plaques and neurofibrillary tau tangles (NFTs) throughout the brain [1–4]. Recent advances in neuroimaging have shown that Aβ plaques and NFTs accumulate in the brain decades before clinical signs of cognitive decline [3, 5–7].

Neuroimaging studies show reduced brain glucose uptake/utilization in AD subjects via fluorodeoxyglucose (*FDG*)-positron emission tomography (PET*)* [5–10]. Furthermore, an overall metabolic deficit in AD subjects, both within the brain and systemically, is apparent [11]. The mitochondrial electron transport chain (ETC) enzyme, cytochrome oxidase (COX, or complex IV), has reduced *V*_max_ (electron flux in the mitochondrial electron transport chain) in AD brain, fibroblasts, and blood [9, 12–23]. AD autopsy brain shows fragmented mitochondrial cristae, and mitochondria vary widely in size compared to age-matched non-demented autopsy brain [24]. In addition to altered mitochondrial morphology, presynaptic terminals show reduced synaptic vesicles and fragmentation of Golgi cisternae in AD postmortem brain [24]. Mitochondrial function is essential for synaptic function and proteostasis, two processes dramatically affected in AD [25–31].

Exercise has been recommended as a nonpharmacological strategy for preventing and mitigating neurodegeneration and cognitive decline [32, 33]. Its efficacy in counteracting the decline in mitochondrial function and modulating key AD pathologies, such as Aβ and NFTs, underscores its therapeutic potential [34]. However, the systemic effects of exercise introduce multifaceted confounders, making interpretation of the effects on AD pathology difficult. Notably, low aerobic capacity has emerged as a recognized risk factor for AD, while high aerobic capacity has been shown to be protective [35, 36]. Moreover, aerobic capacity is a powerful predictor of early mortality in various human populations [37, 38]. Aerobic capacity (also termed cardiorespiratory fitness) is dependent on the capacity of the cardiorespiratory system to supply oxygen to tissues, as well as the capacity for mitochondria in tissues to use oxygen to produce energy. Factors controlling aerobic capacity are linked to genetic factors (intrinsic) and daily physical activity or exercise behavior. Higher activity and regular exercise increase or maintain aerobic capacity during early life and aging, respectively, while sedentary behavior leads to a lower apex for lifetime aerobic capacity and lower aerobic capacity during aging [38].

Rats selectively bred for high vs. low endurance running capacity (treadmill tests to exhaustion during early age) result in two strains of rats with exceptionally different intrinsic aerobic exercise capacities. The high-capacity runner (HCR) and low-capacity runner (LCR) rats display a greater than eightfold difference in intrinsic aerobic capacity in the absence of exercise, providing a platform focused on intrinsic aerobic capacity’s impact on health and avoiding the varying acute and chronic effects of exercise [39]. Moreover, the two strains are a polygenic model of disease that more accurately reflects pathologies driving chronic disease in humans than models with single gene manipulations. LCR rats with low aerobic capacity have previously been shown to be more susceptible to neurodegeneration along with aging-induced loss of mitochondrial function and proteostasis compared to HCR [40, 41]. Most importantly, LCR rats display earlier mortality than HCR rats (28–45% shorter lifespan), demonstrating that the two strains recapitulate the epidemiological findings that aerobic capacity has a pronounced impact on early mortality and chronic disease risk in humans [42]. Leveraging the HCR and LCR rat model presents a valuable opportunity to gain deeper insights into how aerobic capacity influences brain mitochondrial function and AD pathology associated with aging. In this study, we tested the hypothesis that HCR and LCR rats exhibit varied alterations in mitochondrial energetics and AD pathology caused by aging from 18 to 24 months of age. Our data reveal that aging with higher intrinsic aerobic capacity promotes higher brain mitochondrial function that is retained from 18 to 24 months and is also linked to reduced markers of brain AD pathology.

## Methods

### Animal model

The HCR and LCR rat model were developed and characterized at The University of Toledo as previously described [43–45]. All animals were singly housed in a temperature (~ 25 °C) and light-controlled (12:12-h light–dark) room with free access to food and water. Male and female HCR and LCR rats were maintained on a standard chow diet until the age of 18 (male: HCR *n* = 4, LCR *n* = 8; female: HCR *n* = 8, LCR *n* = 8) or 24 months (male: HCR *n* = 5, LCR *n* = 5; female: HCR *n* = 6, LCR *n* = 8). On the morning prior to study, all rats were measured for total body mass. In the morning between 9 and 10 am, male and female HCR and LCR rats, 18 and 24 months of age, were anesthetized at with pentobarbital sodium (100 mg/kg) before being euthanized with exsanguination. Brains were immediately collected, divided into right and left hemispheres, and either utilized for mitochondrial isolation or snap-frozen in liquid nitrogen and stored at − 80 °C. The Institutional Animal Care and Use Committee approved the animal protocols at the University of Kansas Medical Center.

### Mitochondrial isolation

Mitochondria were isolated using a Percoll gradient and ultra-centrifugation. All steps were performed on ice. Briefly, half of the brain was minced with scissors in 1 mL of mitochondria isolation buffer (MIB; 225 mM mannitol, 75 mM sucrose, 6 mM K2HPO4, 1 mM EGTA, 0.1% fatty acid-free BSA, pH 7.2). This mixture was sedimented (using a microfuge) to collect the solid brain tissue. The supernatant was decanted, and the brain tissue was minced again in 1 mL of MIB with scissors. The mixture was sedimented again and the brain tissue re-suspended in 5 mL of fresh MIB. The brain tissue was then homogenized with a Dounce homogenizer, and the resulting homogenate was centrifuged at 1500 × g for 5 min at 4 °C. The supernatant was placed on ice, and the pellet was homogenized in 5 mL of additional MIB and re-centrifuged at 1500 × g for 5 min at 4 °C. This additional supernatant was also placed on ice.

Next, 15%, 23%, and 40% Percoll gradients were made using 100% Percoll in MIB. Of the 40% Percoll gradient 2.3 mL were layered on the bottom of a centrifuge tube, followed by 2.3 mL of 23% Percoll (middle) and 2.3 mL of 15% Percoll (top). Five milliliters of supernatant (from the Dounce homogenate steps) were added to the top of the layered Percoll gradients. Using an SW28.1 Beckman rotor, tubes were centrifuged at 7800 rpm for 13 min at 4 °C. The mitochondrial layer was collected and washed with 8 mL of MIB and centrifuged at 8000 × g for 10 min. Mitochondria were then washed a second time with 8 mL of phosphate-buffered saline (PBS) and re-centrifuged at 8000 × g for 10 min. The mitochondrial pellet was subsequently re-suspended in 400 µL of MIB. Protein assay was completed using a BCA protein assay (ThermoFisher).

### Mitochondrial respiration

A Seahorse XFe96 Analyzer (Agilent Technologies) was used to determine mitochondrial respiratory rates by measuring oxygen consumption rates (OCR). Isolated mitochondria were diluted to 20 µg per 180 µL using MAS buffer (220 mM mannitol, 70 mM sucrose, 10 mM K2HPO4, 5 mM MgCl2, 2 mM HEPES, 1 mM EGTA, 0.2% fatty acid-free BSA). Mitochondria were loaded into seahorse plates at 180 µL per well. The seahorse plate was centrifuged at 1000 × g for 5 min with a brake at 5. The following substrates were successively injected to measure OCR for different respiration rates: (A) 10 mM pyruvate/5 mM malate/4 mM ADP; (B) 2 µM oligomycin; (C) 4 µM FCCP; (D) 10 mM succinate/10 µM rotenone (i.e., complex II-dependent respiration). OCR was measured following a 1-min mix after the addition of injections and measured over 2 min three times. ATP-linked OCR was calculated by state 3 (ADP stimulated) – state 4 (oligomycin stimulated) respiration. Respiratory exchange ratio (RCR) was calculated by state 3 respiration rate ÷ state 4 respiration rate and used to assess mitochondrial coupling control. All data were analyzed using the Agilent Seahorse Wave software.

### Aβ ELISA

Aβ_1-42_ was determined using a rat-specific ELISA from Novus Biologicals. Brain tissue was homogenized in PBS at a 1:9 volume using a glass homogenizer on ice. Samples underwent three freeze thaw cycles and then centrifuged for 5 min at 5000 × g. The supernatant was used in the ELISA per the manufacturer’s instructions. Values were normalized to total protein content determined using a BCA protein assay (ThermoFisher).

### Western blotting

Brain lysates were generated using RIPA buffer with protease and phosphatase inhibitors (Sigma and ThermoFisher). Briefly, an equal amount of protein was resolved via SDS-PAGE on Criterion TGX gels 4–15% (BioRad). Gels were transferred to PVDF membranes and blocked with 5% BSA in PBST. Primary antibodies were incubated overnight at 4 °C followed by three washes with PBST. Secondary antibodies (BioRad) were incubated at room temperature for 1 h. Gels were imaged using WestFemto ECL (ThermoFisher) and a BioRad ChemiDoc XRS imaging system. Loading control was total protein stained using AmidoBlack (Sigma). Primary antibodies were pThr181 Tau and total tau (all antibodies purchased from Abcam).

### Proteomics

Brain lysates were employed for proteomics analysis, following established protocols [46, 47]. In brief, proteins underwent digestion using sequencing-grade modified porcine trypsin (Promega). Subsequently, peptides were separated on an inline 150 × 0.075 mm column packed with reverse phase XSelect CSH C18 2.5 μm resin (Waters), utilizing an UltiMate 3000 RSLCnanosystem (Thermo). Elution was achieved through a 60-min gradient from a 98:2 to 65:35 buffer A:B ratio (buffer A = 0.1% formic acid, 0.5% acetonitrile; buffer B = 0.1% formic acid, 99.9% acetonitrile). The eluted peptides were ionized by electrospray (2.2 kV) and subjected to mass spectrometric analysis on an Orbitrap Exploris 480 mass spectrometer (Thermo).

To generate a chromatogram library, six gas-phase fractions were acquired on the Orbitrap Exploris, with 4 m/z DIA spectra utilizing a staggered window pattern from narrow mass ranges and optimized window placements. Precursor spectra were obtained after each DIA duty cycle, covering the m/z range of the gas-phase fraction. For wide-window acquisitions, the Orbitrap Exploris was configured to acquire a precursor scan followed by 50 times 12 m/z DIA spectra using a staggered window pattern with optimized placements. Precursor spectra were again acquired after each DIA duty cycle.

Proteomic data underwent search using an empirically corrected library, followed by a quantitative analysis to obtain a comprehensive proteomic profile. Identification and quantification were performed using EncyclopeDIA and visualized with Scaffold DIA, incorporating 1% false discovery thresholds at both the protein and peptide levels. The obtained proteomics data were cross-referenced with the MitoCarta3.0 gene inventory to pinpoint proteins with robust evidence of mitochondria localization [48]. Any proteins not included within the MitoCarta3.0 gene inventory were excluded from subsequent analyses. Qiagen’s Ingenuity Pathway Analysis (IPA) (41) was executed to discern changes in mitochondrial pathways. Employing expression core analysis, with the expression log ratio (log-fold change) incorporated for each respective protein, the directionality (*Z*-score) of pathway regulation was determined.

### Statistics

For each group, data were summarized using mean ± SEM. The effect of aging, strain, and their interaction were analyzed by a two-way analysis of variance (ANOVA) within sex, followed by a Fisher’s LSD post hoc analysis when significant interactions were detected. Pearson correlation analysis was used to assess linear relationships. Significant outliers for all data sets were tested and removed using Grubb’s method. All statistical analyses were performed using SPSS statistical software (27.0; SPSS, Inc, IL). Statistical significance was set at *P* < 0.05.

## Results

### Aerobic capacity, age, and sex interact with accumulation of AD pathologies

**Fig. 1:**
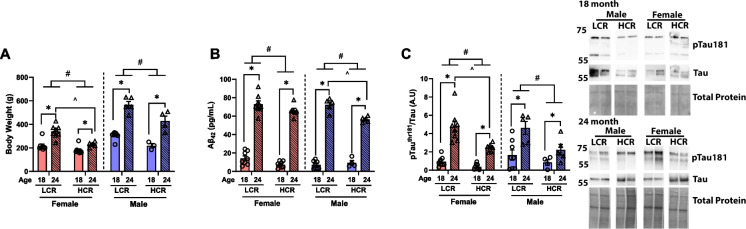
Pathological hallmarks of AD. **A** Body weight (g). **B** Brain amyloid beta (Aβ_42_). **C** Brain phosphorylation of tau ^thr181^ and total tau ratio. Data are presents as means ± SEM. *n* = 4–8/group. * *p* < 0.05 aging effect, # *p* < 0.05 strain effect, ^*p* < 0.05 vs. indicated group

LCR rats display higher body weight compared to their HCR counterparts (main effect of strain, *P* < 0.05, Fig. 1A) which is due to larger overall body size (snout to tail) as reported previously [49]. However, aging resulted in increased body weight in both strains (main effect of age, *P* < 0.05, Fig. 1A).

Aging has been previously associated with increased accumulation of Aβ [50] and phosphorylation of tau (pTau) [51]. In the current study, aging from 18–24 months of age increased the accumulation of Aβ_42_ and pTau (pTau^thr181^/total tau), regardless of strain, in both male and female rats (main effect of age, *P* < 0.05, Fig. 1B and C). Interestingly, HCR rats had reduced accumulation of Aβ_42_ and pTau compared to LCR rats (main effect of strain, *P* < 0.05, Fig. 1B and C). However, the increase in brain Aβ accumulation in male HCR rats from 18 to 24 months was less than the effect seen in LCR rats, an effect not observed in female rats (*P* < 0.05, Fig. 1B). Conversely, the increase in pTau from 18 to 24 months of age in female HCR rats was smaller than that of the LCR rats (*P* < 0.05, Fig. 1C), a trend not observed in male rats. These findings reveal that aerobic capacity impacts development of AD brain pathologies but that the effects are impacted by sex and whether the AD pathology marker is Aβ or pTau.

### Aerobic capacity, age, and sex influence brain mitochondrial energetics

**Fig. 2:**
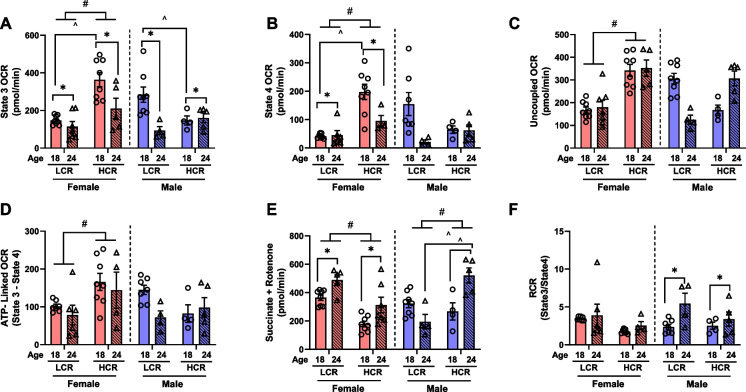
Brain mitochondrial respiration. **A** ADP-stimulated state 3 O_2_ flux. **B** Oligomycin-stimulated state 4 O_2_ flux. **C** FCCP-stimulated uncoupled O_2_ flux. **D** ATP-linked O_2_ flux. **E** Complex II supported O_2_ flux. **F** Respiratory control ratio (RCR). Data are presents as means ± SEM. *n* = 4–8/group. *p < 0.05 aging effect, #*p* < 0.05 strain effect, ^*p* < 0.05 vs. indicated group

Defects in mitochondrial energetics in the brain is a hallmark characteristic associated with AD [9, 12–23]. Aging from 18 to 24 months significantly reduced brain mitochondrial state 3 and state 4 respiration in female rats, irrespective of strain (main effect of age, *P* < 0.05, Fig. 2A and B). Female HCR rats exhibited heightened mitochondrial state 3, state 4, and uncoupled respiration compared to their LCR counterparts (main effect of strain, *P* < 0.05, Fig. 2A–C) at both the 18- and 24-month timepoints. However, this effect was largely driven by the significantly greater mitochondrial state 3 and state 4 respiration in 18-month-old female HCR rats that had far higher levels than LCR counterparts (*P* < 0.05, Fig. 2A and B). When excluding the mitochondrial state 3 difference, female HCR rats demonstrated increased ATP-linked respiration compared to female LCR rats (main effect of strain, *P* < 0.05, Fig. 2D). In contrast, aging from 18 to 24 months increased complex II-supported respiration in female rats, independent of strain (main effect of age, *P* < 0.05, Fig. 2E). However, female HCR rats exhibited lower complex II respiration than female LCR rats (main effect of strain, *P* < 0.05, Fig. 2E). No differences in RCR were observed among female rats (Fig. 2F).

Similarly, aging from 18 to 24 months reduced mitochondrial state 3 respiration in male rats, regardless of strain (main effect of age, *P* < 0.05, Fig. 2A). Notably, male HCR rats exhibited decreased state 3 respiration at 18 months old compared to their LCR counterparts (*P* < 0.05, Fig. 2A). However, no differences were observed in state 4, uncoupled, and ATP-linked respiration between strains in male rats (Fig. 2B–D). Male HCR rats demonstrated greater complex II respiration compared to male LCR rats (main effect of strain, *P* < 0.05, Fig. 2E). This effect was primarily driven by 24-month-old male HCR rats, displaying heightened complex II respiration compared to 24-month-old male LCR rats (*P* < 0.05, Fig. 2E). Interestingly, aging increased the RCR across all male rats, irrespective of strain (main effect of aging, *P* < 0.05, Fig. 2F). These findings highlight the intricate interplay between aerobic capacity, sexual dimorphism, and brain mitochondrial function associated with aging.

### Brain mitochondrial respiration is associated with AD pathologies

**Fig. 3:**
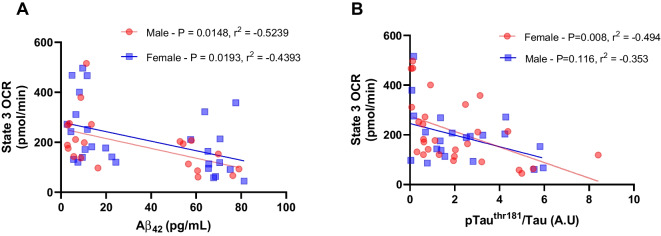
Brain mitochondrial respiration associated with pathological hallmarks of AD. **A** Correlation between brain state 3 O_2_ flux and Aβ_42_. **B** Correlation between brain state 3 O_2_ flux and phosphorylation of tau ^thr181^ and total tau ratio. Data are presents as means ± SEM. *n* = 4–8/group

The correlation analysis, considering all groups separated by sex, revealed a negative association between brain state 3 respiration and Aβ_42_ levels in male (*P* = 0.0148, *r*^2^ =  − 0.5239) and female rats (*P* = 0.0193, *r*^2^ =  − 0.4393, Fig. 3A). Similarly, there was a negative association between brain state 3 respiration and pTau levels in female rats (*P* = 0.008, *r*^2^ =  − 0.484, Fig. 3B), a relationship absent in male rats. These findings suggest a potential link between the decline in mitochondrial respiration and AD pathology or vice versa.

### Aerobic capacity and sex influence the brain mitochondrial proteome

**Fig. 4:**
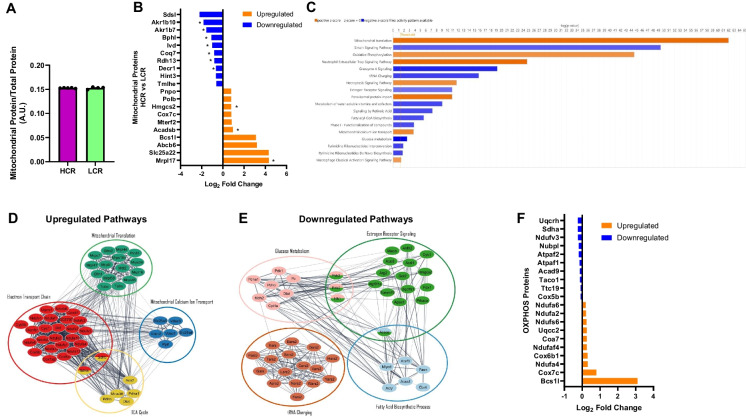
Female brain mitochondrial proteomics at 18 months of age. **A** Mitochondrial protein abundance. **B** Top 10 upregulated and downregulated mitochondrial proteins based on Log2-fold change. **C** IPA analysis of the mitochondrial proteome. **D** String pathway of proteins in upregulated pathways. **E** String pathway of proteins in downregulated pathways. **F** OXPHOS top 10 upregulated and downregulated mitochondrial proteins based on Log2. *n* = 4–5. *p < 0.05 vs. LCR

**Fig. 5:**
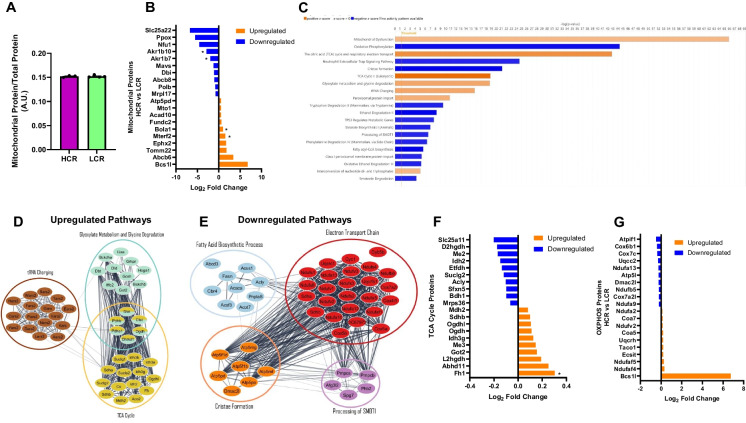
Male brain mitochondrial proteomics at 18 months of age. **A** Mitochondrial protein abundance. **B** Top 10 upregulated and downregulated mitochondrial proteins based on Log2-fold change. **C** IPA analysis of the mitochondrial proteome. **D** String pathway of proteins in upregulated pathways. **E** String pathway of proteins in downregulated pathways. **F** TCA cycle top 10 upregulated and downregulated mitochondrial proteins based on Log2. **G** OXPHOS top 10 upregulated and downregulated mitochondrial proteins based on Log2. *n* = 3–4. *p < 0.05 vs. LCR

To investigate potential proteins and pathways that may promote differences in AD pathologies due to aerobic capacity, we used untargeted proteomics on whole brain homogenates from 18-month-old HCR and LCR rats (*n* = 3–5/group). We only performed the analysis on the 18-month group to shed light on the transition that occurred from 18 to 24 months across both strains. We then cross-referenced the identified proteins with MitoCarta3.0 to extract known mitochondrial-specific proteins. In total, 3879 and 4040 total proteins were identified in whole brain homogenates, with 588 and 592 identified as mitochondrial proteins in females and males, respectively. The summed protein abundance of mitochondrial proteins (MitoCarta3.0) to total protein abundance revealed no differences in total mitochondrial protein content between strains (Figs. 4A and 5A), indicating that mitochondrial proteome differences are not influenced by overall mitochondrial content.

In the female mitochondrial proteome, 19 mitochondrial proteins exhibited significant differences between HCR and LCR rats (*P* < 0.05). HCR females had increases in proteins associated with mitochondrial translation (Mrpl17), metabolism of fatty acids or branched-chain amino acids (Acadsb), and ketone metabolism (Hmgcrs2) compared to female LCR rats (*P* < 0.05, Fig. 4B). Additionally, female HCR rats showed a reduction in proteins associated with aldo–keto reductase family (Akr1b10, Akr1b7), leucine metabolism (IVD), and fatty acid metabolism (Decr1) (*P* < 0.05, Fig. 4B). IPA analysis of all mitochondrial proteins revealed that female HCR rats had increases in proteins associated with mitochondrial translation, oxidative phosphorylation (OXPHOS), and mitochondrial calcium ion transport, along with a reduction in sirtuin signaling, glucose metabolism, estrogen receptor signaling, and fatty acid biosynthetic processes compared to LCR (Fig. 4C). Interestingly, several proteins involved in these upregulated and downregulated pathways interacted, suggesting potential crosstalk between these pathways (Fig. 4D and E). When examining the oxidative pathway specifically, several upregulated proteins involved in complex I (Ndufa4, Ndufaf4, Ndufs6, Ndufa2, Ndufa6) and complex IV (Cox7c, Cox6b1, Coa7) were identified when comparing female HCR rats to female LCR rats (Fig. 4F), indicating their potential role in influencing the differences in female mitochondrial respiration previously shown (Fig. 2).

In the male brain mitochondrial proteome, 20 mitochondrial proteins exhibited a significant difference between HCR and LCR rats (*P* < 0.05). HCR males had an increase in proteins associated with the regulation of mitochondrial transcription (Mterf2) and mitochondrial iron–sulfur cluster (Bola1) and a reduction in aldo–keto reductase family proteins (Akr1b10, Akr1b7) (*P* < 0.05, Fig. 5B) compared to LCR. IPA analysis of the mitochondrial proteome revealed that male HCR rats exhibited an increase in proteins associated with the tricarboxylic acid (TCA) cycle, glyoxylate metabolism, glycine degradation, and tRNA charging, along with a decrease in proteins associated with OXPHOS, cristae structure, and fatty acid biosynthetic process (Fig. 5C). Several proteins from these upregulated and downregulated pathways interacted with one another, suggesting potential mutual influence (Fig. 5D and E). The increase in the TCA cycle is influenced by the increase in several dehydrogenase proteins (FH1, Idh3g, Ogdh, Sdhb, Mdh2) (Fig. 5F). However, the increase in TCA cycle is countered by the reductions in complex IV (Cox6b1, Cox7c, Cox7a2l) and complex V proteins (Atpif1, Atp5l) (Fig. 5G), which may influence the reduction in mitochondrial respiration at 18 months found in male HCR rats (Fig. 2). Therefore, the mitochondrial proteome at 18 months of age may be essential for mitochondrial function, and aerobic capacity in females may have a greater influence on the mitochondrial proteome, promoting protection against AD pathology. In contrast, in males, the mitochondrial proteome and function may not be the mechanism by which aerobic capacity protects against AD pathology due to a smaller number of differences in mitochondrial proteome findings between strains.

## Discussion

A decline in brain mitochondrial function (i.e., reduced respiration, increased H_2_O_2_ emission, altered Ca^2+^ handling), alongside increased levels of Aβ and NFTs, represents prevalent abnormalities associated with aging [52–54]. Moreover, these mitochondrial energetic defects have previously been associated with advanced cognitive decline and the progression of AD [9, 12–23]. Exercise has demonstrated efficacy in mitigating the decline in mitochondrial function and alterations in Aβ and NFTs, contributing to reduced cognitive decline in aging [34]. However, the systemic effects of exercise introduce multifaceted factors that may impact brain mitochondria and AD pathologies, complicating mechanistic understanding. While high vs. low intrinsic aerobic capacity has been previously linked to cognition and brain mitochondrial function, the precise impact of intrinsic aerobic capacity on aging-induced changes in brain mitochondrial respiration and links to AD pathology remains unclear [40]. This study aimed to investigate whether disparities in intrinsic aerobic capacity influence brain mitochondrial function, brain mitochondrial proteome, and markers of AD in response to aging from 18 to 24 months of age, a period of time that is equivalent to 45 and 60 years in human health [55]. The results demonstrate that lower intrinsic aerobic capacity is linked to increased Aβ and pTau levels with aging. Additionally, lower aerobic capacity is associated with an earlier decline in mitochondrial respiration during aging, particularly in female rats compared to their male counterparts. We also found that brain mitochondrial respiration correlates with AD-associated pathologies in rats with high or low intrinsic aerobic capacity. We also highlight that higher aerobic capacity alters the mitochondrial proteome related to OXPHOS and the TCA cycle at 18 months of age, potentially laying the foundation for maintaining mitochondrial respiration rates. Overall, these data suggest that lower aerobic capacity promotes a more pronounced reduction in mitochondrial function and an increase in AD-associated pathologies, potentially contributing to cognitive decline, as previously observed in LCR rats [40].

Aging is associated with a decline in brain mitochondrial enzymatic activity and respiration [52–54]. Additionally, impairments in brain mitochondrial function manifest as early characteristics in the development of AD [9, 12–23]. Exercise training has been demonstrated to counteract the decline in mitochondrial respiration and mitigate oxidative stress in the brain [56]. Similarly, higher intrinsic aerobic capacity promotes greater mitochondrial respiration in the brain [40]. Our study adds further confirmation that aerobic capacity modulates brain mitochondrial function by promoting higher mitochondrial respiration with aging. However, irrespective of aerobic capacity, aging results in a reduction in brain mitochondrial respiration. As a result, exercise and aerobic capacity may offer protection by establishing early differences in the mitochondrial proteome and function that contribute to maintaining greater function throughout aging. Our data indicate distinct differences at 18 months of age in OXPHOS and the TCA cycle. Additionally, we have previously reported heightened brain mitochondrial protein synthesis in 24-month-old LCR rats compared to their HCR counterparts in both males and females [41]. This suggests that at 18 months, differences in aerobic capacity enhance mitochondrial function through alterations in the proteome, reducing the compensatory mitochondrial protein synthesis and mitigating deficits in mitochondrial respiration associated with aging [40, 41]. Future studies are needed to confirm whether differences in the mitochondrial proteome serve as the driving force in maintaining mitochondrial function and providing protection against AD pathology.

Sex differences play a significant role in the diagnosis of AD, with higher rates observed in females. In the context of AD, females exhibit a more pronounced downregulation of ATP subunits and cytochrome oxidase in the brain than males, suggesting females may be more susceptible to mitochondrial dysfunction in the brain [57]. Our data reveal a more substantial impact of low aerobic capacity on brain mitochondrial respiration in female rats than the effects found in males. These findings underscore the importance of considering sex-specific responses when exploring relationships between aerobic capacity, mitochondrial function, and the pathogenesis of AD. The cause of the sexual dimorphism in brain metabolism may be related to changes in ovarian function and estrogen signaling, which has positive effects on mitochondrial function in young females, but after aging-induced changes in ovarian function, reduced estrogen signaling can lead to compromised mitochondrial function [58].

Physically active women experience cognitive improvement and a reduced risk of dementia [59, 60]. Similarly, we found that higher aerobic capacity protects against AD pathology. This reduced risk may be from aerobic capacities ability to increase OXPHOS proteins, particularly in complex I protein abundance. Interestingly, in postmortem brain tissue of female subjects, proteomics reveals a downregulation in complex I protein abundance in early- and late-stage AD [61]. These changes in the mitochondrial proteome may result from the regulation of mitochondrial protein synthesis. Our proteomic data showed that enhanced aerobic capacity promotes a greater abundance of mitochondrial translation proteins, which are linked to several proteins in the electron transport chain. Alterations in mitochondrial translation have been reported to result in disordered mitochondrial protein synthesis and a reduction in mitochondrial respiration [62]. Therefore, it may be essential for females to maintain proper mitochondrial translation of OXPHOS proteins to preserve mitochondrial function and prevent the onset of AD pathology. In contrast, male HCR rats exhibit reduced mitochondrial respiration and OXPHOS proteins but maintain mitochondrial respiration with aging, resulting in a reduction in the development of AD pathology compared to male LCR rats. Thus, male HCR rats may employ different mechanisms than female HCR rats, which are not apparent in this dataset. Exploring an aging proteomic dataset in future studies may provide valuable insights into these mechanisms.

Physical activity has been demonstrated to reduce levels of Aβ and pTau in both humans and rodents [34]. Consistent with these findings, our results indicate that higher intrinsic aerobic capacity is associated with lower Aβ and pTau, particularly in the context of aging. Mitochondrial function correlates with Aβ production and localization within mitochondria [63–65]. Notably, previous research has identified the accumulation of pTau in mitochondria of aged LCR rats but not in HCR rats [40]. Interestingly, we report an association between mitochondrial function and Aβ and pTau levels, irrespective of strain. The precise origins of the Aβ and pTau accumulation remain unclear, whether from an increase in production, a reduction in clearance, or a combination of both. However, these data suggest that maintaining a higher aerobic capacity minimizes the accumulation of Aβ and pTau associated with aging, potentially offering protection against AD.

Our study has certain limitations. While mitochondria play a crucial role in cellular energy metabolism, intracellular Ca^2+^ handling, redox state (i.e., H_2_O_2_ emission), and apoptosis signaling, the scope of our investigation was confined to mitochondrial respiration as a marker of mitochondrial function, specifically ATP production. This, however, does not offer a comprehensive assessment of overall mitochondrial functionality in the HCR and LCR rats, as several other vital mitochondrial-mediated events may be altered or impaired with aging, sex, and AD pathologies [66]. Furthermore, a previous study has observed cognitive decline and loss of hippocampal volume in LCR rats [40]. Although our primary focus centers on age-related changes, an exploration of the long-term effects and functional correlations with cognitive decline or other behavioral outcomes would contribute to a more thorough understanding of the observed findings.

## Conclusion

In conclusion, our study leveraging a novel rat model of high vs. low aerobic capacity demonstrates that higher intrinsic aerobic capacity elevates mitochondrial respiration and reduces AD pathologies. Notably, our findings underscore a more pronounced effect of aerobic capacity on brain mitochondrial respiration and proteome in female rats. Future investigations should aim to delve deeper into the intricate molecular and cellular mechanisms underpinning these observed effects.

## Data Availability

Data are available upon request to the corresponding author.
